# Innovative molecular and immunological approaches of heterophyiasis infecting some Egyptian marketed fishes

**DOI:** 10.1186/s12917-024-04226-1

**Published:** 2024-08-30

**Authors:** Olfat A. Mahdy, Reem M. Ramadan, Mai A. Salem

**Affiliations:** Department of Parasitology, Faculty of Veterinary Medicine, Cairo University, Giza, 12211 Egypt

**Keywords:** Heterohyid flukes, PCR, Genetic characterization, Gene expression *centrocestus*, *Heterophyes*

## Abstract

Heterophyiasis is a highly endemic disease in the Nile Delta, Egypt, where people consume raw or undercooked *Oreochromis niloticus* and *Mugil cephalus*. Birds and rats play a crucial role in fish-borne zoonotic trematode transmission since they serve as natural and experimental hosts. This study aimed to update the epidemiological information, morphological description, molecular identification and gene expression of two distinct heterophyid metacercariae in Giza, Wadi Al-Rayan, and Lake Manzala, Egypt, whereas various heterophyid infections could be expected. The present *Centrocestus formosanus*,* Heterophyes heterophyes*, and *Heterophyes nocens* with accession numbers OR947651.1, OR947700.1, and OR947719.1, respectively, matched with those recorded in the GenBank. Findings of the current investigation indicated that various cytokines like IL-1β, MHC-II, and TNF-α rapidly elevated in the infected pigeon’s intestines. Additionally, the infection expanded due to the parasite’s ejection from the host and the host’s clinical affliction, which induced humoral immune responses. Interestingly, investigation of other trematode species is in extreme demand in terms of zoonoses. We suggest controlling snails, managing migratory birds, and examining and frying fishes to the point when the encysted metacercariae is destroyed.

## Introduction

Worldwide public awareness of fish-borne zoonotic trematodes is growing with over 18 million individuals afflicted with these infections. Severe infections can produce mucosal damage and certain gastrointestinal symptoms in humans [1] and fish-eating birds [2]. It has been documented that both wild and farmed fishes in Egypt contain heterophyid encysted metacercaria [3]. In the Nile Delta of Egypt, where eating raw or undercooked mullet or pickling mullets is a frequent practice, heterophyid is a highly prevalent disease [4, 5]. Snails serve as the first intermediate hosts for trematodes that use mullets as secondary intermediate hosts, while piscivorous birds serve as the final hosts [6]. According to [7–9], the major threat to the global spread of parasitic disorders caused by the causative agent helminth is the large number of infected hosts, including humans. Furthermore, humans quickly acquire infection in locations where fish is usually ingested raw, such as some local districts of the Nile Delta. [10].

In addition, *Centrocestus formosanus* (*C. formosanus*), is a digenean trematode that is a member of the *Heterophyidae* family and needs three distinct hosts to complete its life cycle. It uses fishes (*Oreochromis niloticus*) and snails (*Melanoides tuberculata*) as first and second intermediate hosts, respectively, to finish its life cycle, with mammals serving as definitive hosts [11]. Several farmed fish species have *C. formosanus* encysted metacercariae (EMC) encyst in their gills, leading to severe pathological changes in the gill architecture [12]. These changes may exacerbate respiratory issues, lower productivity, and mortalities in young fishes. Due to eating raw or undercooked fishes that contain EMC, zoonotic infections related to *C. formosanus* have been reported globally, notably in the Egyptian Nile Delta and in European and Asian nations [12].

When immune cells are triggered by many pathogens, including bacterial, viral, or parasite components, they release cytokines. They can alter immune responses in an autocrine or paracrine way when they bind to the right receptors. Examples of stimulating agents include interleukins, interferons, and tumor necrosis factors (TNFs) [13]. For innate immunity to eradicate infections and attract neutrophils, lymphocytes, and macrophages to the affected areas, IL-1, IL-10, IFN-γ, and TNF-α are required. A crucial element of adaptive immunity is the gene set known as the major histocompatibility complex (MHC). Encoding cell-surface glycoproteins is the primary role of MHC class II genes [14].

In Egypt, heterophyiasis is not a well-known public health concern, despite being a severe infection. This study aimed to update the epidemiological data in two distinct heterophyid EMC in Egypt: Giza, Wadi Al-Rayan, and Lake Manzala. This is because *Oreochromis niloticus (O. niloticus)* and *Mugil cephalus* (*M. cephalus*) are associated with various forms of heterophyid infection in these regions. This study aimed to identify the heterophyid-encysted metacercariae that infect *O. niloticus* and *M. cephalus* by using morphological and genetic techniques on recovered adult flukes from experimentally infected pigeons. Pigeons infected with *Heterophyes (H.)* species are assessed for humoral immune responses using quantitative real-time polymerase chain reaction (qRT-PCR).

## Materials and methods

### Fishes sampling

A total of 280 fishes, 200 of *O. niloticus* and 80 of *M. cephalus*. The fishes were gathered from three separate locations in Egypt: Lake Wadi Al-Rayan (the Fayoum province), the River Nile (the Giza province), and Lake Manzala (the Dakahlia province) during the period from January to December 2023. The fishes were shipped in plastic bags with air inside and roughly 30% of the fish’s volume was filled with water. In the meantime, the recently deceased fishes were labeled, placed in sterile plastic bags, chilled in an ice box at 4 °C, and transported right away to the lab for analysis [15].

### Detection, identification and collection of heterophyid encysted metacercariae

Fishes were examined microscopically after compressed gill arch and very small pieces of the fish muscles by using compresorium for the presence of the EMC in gills and muscles of different regions. The distribution of EMC in the different fish body parts (head, trunk, tail, and viscera) was studied from the investigated geographical localities. The EMC was microscopically measured using a light microscope (Olympus microscope (CX41, Japan) using an objective lens (4×&10×) magnification power [16]. The morphometric identification of EMC was then performed by studying the characteristic features, such as the shape of the cyst, suckers, and excretory bladder according to [6, 17].

Fish tissues that were infected with EMC were collected, and the fishes were artificially digested for one hour at 37 °C in a vibrating water bath using 0.25% pepsin in 0.85% NaCl. After being filtered via sieves, the digested tissues precipitated in 0.85% NaCl. Using a binocular microscope, the identified EMC were divided according to their morphological characteristics [6]. One experimental subject was infected with each type of EMC that was gathered [3]. Under a dissecting microscope, distinct morphological types of EMC were gathered in individual tubes and counted before being removed with a Pasteur pipette. The cysts were stored at 4 °C in a 0.75% saline solution until they were utilized to infect laboratory pigeons and rats in an experiment.

### Experimental design

All experimental animals were obtained from the laboratory animal breeding unit (Department of Animal and Poultry Management and Behavior, Faculty of Veterinary Medicine, Cairo University) and examined to be free from parasitic infections and divided into two groups; the first group includes pigeons infected with *Heterophyidae* EMC from the muscles of *M. cephalus*. The second one includes rats infected with *Centrocestus* EMC from the gills of *O. niloticus.* Each group is subdivided into two subgroups (infected and control group).

#### Experimental infection of pigeons with heterophyid EMC from *M. cephalus*

Twenty healthy one-week-old *Columba livia domestica* pigeons were raised individually in dedicated cages weighing 45–65 g using the traditional method. To demonstrate that the feces were clear of parasite infections prior to the experimental infection, they were collected and analyzed every day for three days in a row. Using a Pasteur pipette, an infectious dosage between 300 and 400 *Heterophyidae* EMC was given to each pigeon in the infected subgroup (*n* = 10) (Table 1). The control subgroup (*n* = 10) that was remained uninfected. The pigeons were kept apart and given unlimited access to water and grain [14].

#### Experimental infection of laboratory rats with *Centrocestus* EMC from *O. Niloticus*

Twenty rats grew up at Cairo University’s Animal House, Cairo University Department of Parasitology, Faculty of Veterinary Medicine. They were split up into two subgroups, with ten rats apiece (G). Using a Pasteur pipette, an infectious dosage between 100 and 150 *Centrocestus* EMC from the gills of *O. niloticus* was given to each rat in the infected subgroup (Table 1). A control group that was not affected was the second one. The rats were kept in separate housing groups and given unlimited access to water and corn [11].

**Table 1:** Experimental design of laboratory animals with different obtained EMC

Experimental animal	Dosage of EMC	Type of recovered adult fluke
Pigeon (*n* = 10)	300–400 *Heterophyes* spp. EMCs from muscles of *M. cephalus*	*H. heterophyes* *H. nocens*
Rats (*n* = 10)	100–150 *C. formosanus* EMC from gills of *O. niloticus*	*C. formosanus*

On the seventh day following infection, the infected rats and pigeons were dissected, and their small intestines were opened longitudinally in a Petri dish full of phosphate buffer saline (pH 7.40), allowing them to be inspected for acquired worms using a stereomicroscope (Olympus, SZ61, and CX41 microscope, Japan) [2]. Using a Pasteur pipette, the identified trematodes were gathered in normal saline, counted, and preserved in 10% formalin in a little vial. For fresh preparation, adult worms were collected [18]. Based on earlier research, the morphological identification of the flukes that were found was done [19]. Drops were taken daily from each group and analyzed using the flotation and sedimentation method in order to estimate the prepatent period [20, 21]. The worms that were obtained were permanently mounted using the methods described by [22, 23].

### Molecular identification

#### PCR amplification

Extraction of genomic DNA 30 selected specimens of each trematode species was selected for molecular characterization. Genomic DNA was extracted using a DNA Purification Kit (GeneJET; Fermentas Life Sciences, Lithuania), following the protocol of the manufacturer. The extracted DNA was stored in a deep freezer at − 80 °C until use. According to [24], the universal primers (Forward: 5′ AGGAATTCCTGGTAAGTGCAAG 3′) and (Reverse: 5′ CGTTACTGAGGGAATCCTGG- 3′) were used to amplify partial 28 S rRNA (large subunit ribosomal RNA) gene by PCR. The MyTaqTM Red Mix kit (Bioline) protocol was followed in conducting the PCR reactions. Starting with a five-minute initial denaturation at 95 °C, the PCR process proceeded through 35 cycles of denaturation at 95 °C for thirty seconds, annealing at 57 °C for thirty seconds, and extension at 72 °C for thirty seconds, culminating in a final ten minutes of extension at 72 °C [3]. Following electrophoresis in an agarose gel, PCR amplicons were stained with ethidium bromide and seen using a UV transilluminator [25].

#### DNA sequencing

PCR amplicons were purified using the QIAGEN Extraction Kit in accordance with the manufacturer’s instructions. The purified amplicons were sent to the Animal Health Research Institute in Egypt to be sequenced using the Big Dye Terminator v3.1 cycle sequencing kit chemistry, using the identical primers that were used for PCR amplification. The DNA sequencing procedures were electrophoresed using ABI Prism 3700 DNA Analyzers. The obtained sequences were analyzed and changed using Bio Edit [26]. The 28 S rRNA assembled sequences of *Centrocestus* and *Heterophyes* species were aligned with other 28 S rRNA regions of trematodes that were available in GenBank using nucleotide BLAST (https://blast.ncbi.nlm.nih.gov/Blast.cgi). Following that, the sequences were added to the GenBank database and assigned accession codes (OR947651.1, OR947700.1, and OR947719.1). Using MEGA 11, the neighbor-joining model was used to conduct a phylogenetic analysis of the assembled sequences [27]. The following parameters were applied: substitutions: transversions and transitions, constant rate of variation between sites, and homogeneous pattern among lineages, using 1000 bootstrap replicates.

### Immunological expression of cytokines by quantitative real-time polymerase chain reaction (qRT-PCR)

#### Sampling

Aseptic dissection was used to obtain intestinal samples from pigeons that had been subjected to *Heterophyes* EMCs during the experiment. Five intestinal samples from uninfected pigeons were collected in the same manner as negative controls.

#### Isolation of RNA

Following the manufacturer’s instructions, a total RNA isolation kit (Ambion, Applied Biosystems) was used to isolate the mRNA from 100 mg of the studied samples that were all present in the infected samples and selected for expression analysis. Lysing Matrix D tubes (MP Biomedicals) were used to homogenize the tissues in a FastPrep-24 homogenizer. The quantity and quality of the generated mRNA were evaluated with Nanodrop (Thermo Scientific). Reverse transcription was performed using the High-Capacity cDNA Archive Kit (Applied Biosystems) according to the manufacturer’s instructions [15].

#### The protocol of the qRT-PCR

The sequences of pigeons deposited in the GenBank and listed in Table 2 served as the primary basis for these primers. In addition, to create cDNA, 25, 2 µL of RNA extract was mixed with a hexamer primer solution. After five minutes of incubation at 65 °C in a thermal cycler, the mixture was immediately placed on ice for at least one minute. Furthermore, reverse transcriptase (2 µL), 10 mM MgCl2, and 1 mM dNTPs were added to ten microliters of a first standard reaction (2×), which were then incubated for ten minutes at 25 °C [28].

**Table 2:** Primer sets for qRT-PCR analysis of the impact of trematode infection on cytokine expression in pigeons

Primers	Sequence (5ʹ–3ʹ)	References
**IL-1β**	F-TGGCCCTGACTGAACCACTG R-TCAGACCCACGCCACAGAAC	[29]
**MHC-II**	F-ATGTCCAAGCTGCTGAAGATT R-TGCCGTCTGACTTCTTCACC	[30]
**TNF-α**	F-GCGCAGTCTGTCATTGGTT R-ACTGGACACGCTCACTGTAGTG	[31]
**β-actin**	F-GGCTACTCCTTCACCACCACAG R-GGGCAACGGAACCTCTCATT	[15]

### Statistical analysis

The findings are shown as means ± standard deviation [32]. One-way ANOVA analysis revealed significant differences in the tested values. Software from SPSS Inc. (version 27.0) was used for all statistical analyses, with a significance level of *p* < 0.05 [33, 34].

## Results

### Epidemiology of EMC in the investigated fishes

In the present study, the total prevalence of the investigated fishes infected with EMC showed that 143 out of 280 samples (51.07%) were infected. The rate of metacercarial infection in *O. niloticus* collected from Wadi Al-Rayan and River Nile was 65% and 51%, respectively. While *M. cephalus* of Lake Manzala metacercarial infection was 33.75% (Table 3).

**Fig. 1:**
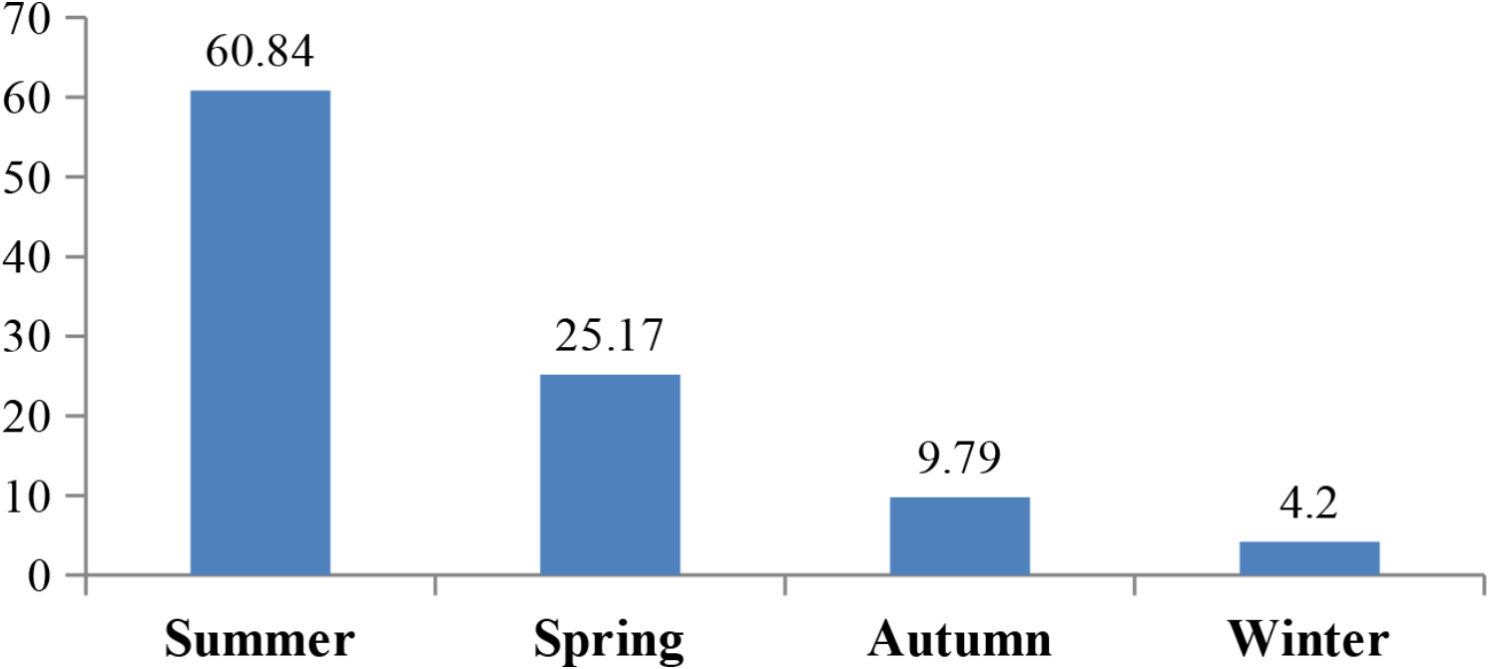
Seasonal variation of EMC in the investigated fish spp

**Fig. 2:**
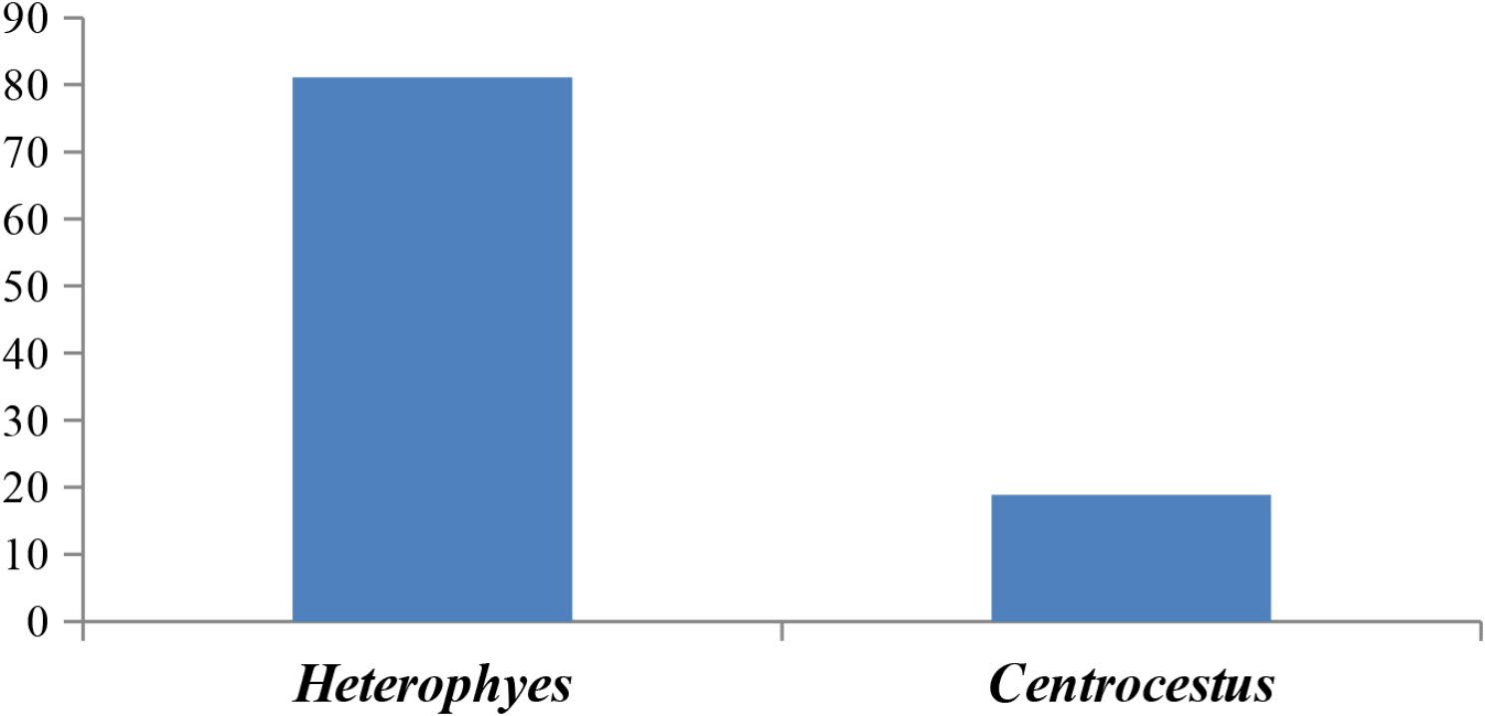
Seasonal variation of EMC in the investigated fish spp

The study found that there was a significant seasonal variation in the prevalence of EMC infection (*p* = 0.0038). Summer had the greatest infection rate at 60.84 ± 7.42, followed by spring, autumn, and winter at 25.17 ± 3.73, 9.79 ± 0.72, and 4.2 ± 0.22, respectively (Fig. 1). *Heterophyes* sp. was the most common cyst among the infected fishes reported in this study (81.12 ± 6.56), while *Centrocestus* sp. had the lowest prevalence (18.89 ± 2.71), according to the aggregation index of all observed EMC (Fig. 2). *Heterophyes* and *Centrocestus* spp. were the cyst species that were recorded in the provinces of Giza and Fayoum, although *Heterophyes* spp. were only recorded in the province of Dakahlia. Regarding the distribution of encysted metacercaria in the various fish body areas, the head region of *M. cephalus* (3.5 ± 1.13) had the lowest number of cysts per gram of tissue, while the tail region of *O. niloticus* (15.5 ± 3.72) had the highest number and the lowest was in the head region of *M. cephalus* (3.5 ± 1.13).

**Table 3:** Prevalence of EMC among fish spp. from different localities

Fish species	O. niloticus		M. cephalus
**Locality**	Wadi Al-Rayan	River Nile	Lake Manzala
**Prevalence**	65.00%	51.00%	33.75%
**Total**	143/280 (51.07%)		

### Experimental infection of pigeons and rats with heterophyid EMC

Based on the verified and recognized adult flukes that were recovered from pigeons and rats following experimental infection by EMC derived from *M. cephalus* and *O. niloticus*, the exact identification of these cysts was determined. This finding identified three flukes: *H. heterophyes* and *H. nocens* from the intestines of pigeons (fed on infected muscles) and *C. formosanus* from the intestines of rats (fed on infected gills). Their eggs had appeared 5–8 days after infection, according to fecal testing.

### Morphological identification of the EMC and adult flukes

**Fig. 3:**
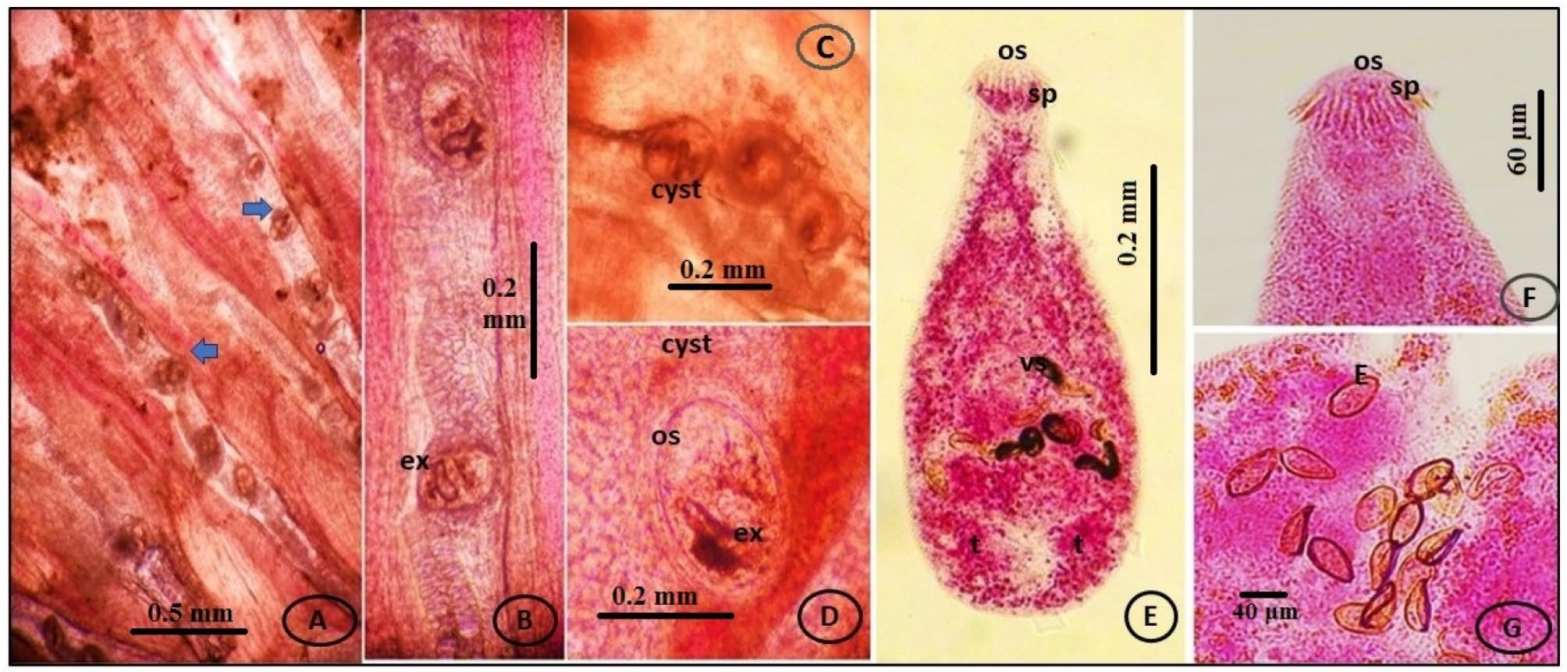
(**A**-**B**) The encysted metacercariae of *C. formosanus* infected gills filaments of Nile Tilapia (*O. niloticus*). (**C**-**D**) high magnification of EMC shows the y-shaped dark excretory bladder(ex), oral sucker armed (os)with spines (sp.), (**E**) An adult worm recovered from rats 7 days after infection, stained with acetocarmine. (**F**) Oral sucker of an adult worm with circumoral spines. (**G**) Eggs showed in the uterus fluke

**Fig. 4:**
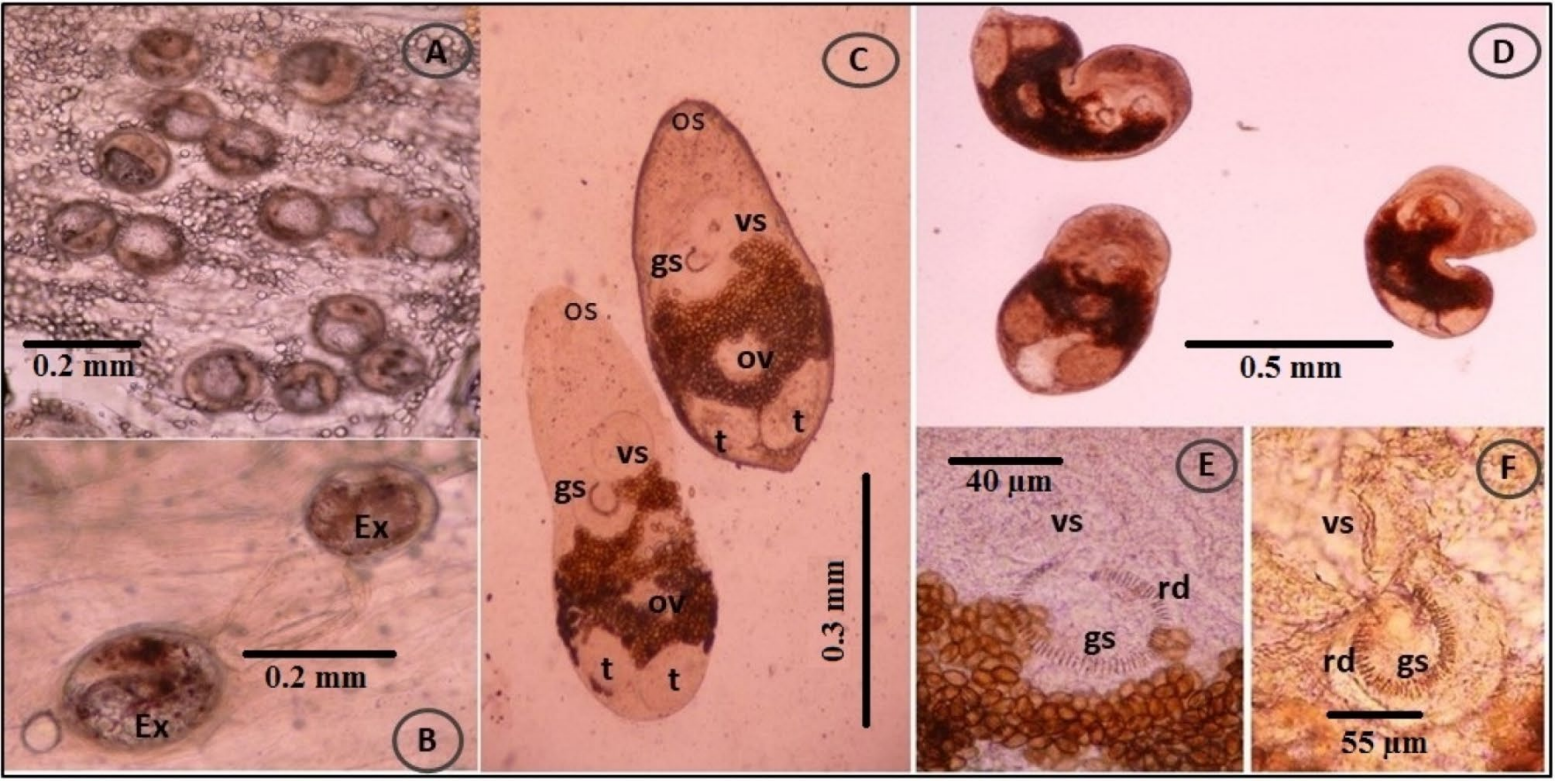
(**A**-**B**) Fresh specimens of *Heterophyes* species EMC collected from *M. cephalus* show spherical to elliptical in shape, with a cyst wall and a crescent-shaped excretory bladder (Ex). (**C**-**D**) Adult flukes; *H. heterophyes* and *H. nocens* collected from the small intestine of experimentally infected pigeons. Noted that oral sucker (os), ventral sucker (vs.) and genital sucker (gs), two horizontal testes (t), ovary (ov). (**E**) *H. heterophyes* high magnification of armed gonotyl, lying to the dextro-posterior margin of the ventral sucker, armed with more than 70 chitinous spines (rodlets = rd) around the genital sucker (gs). (**F**) *H. nocens* high magnification of armed gonotyl, lying to the dextro-posterior margin of the ventral sucker, armed with less than 70 chitinous spines (rodlets = rd) around the genital sucker (gs)

**Fig. 5:**
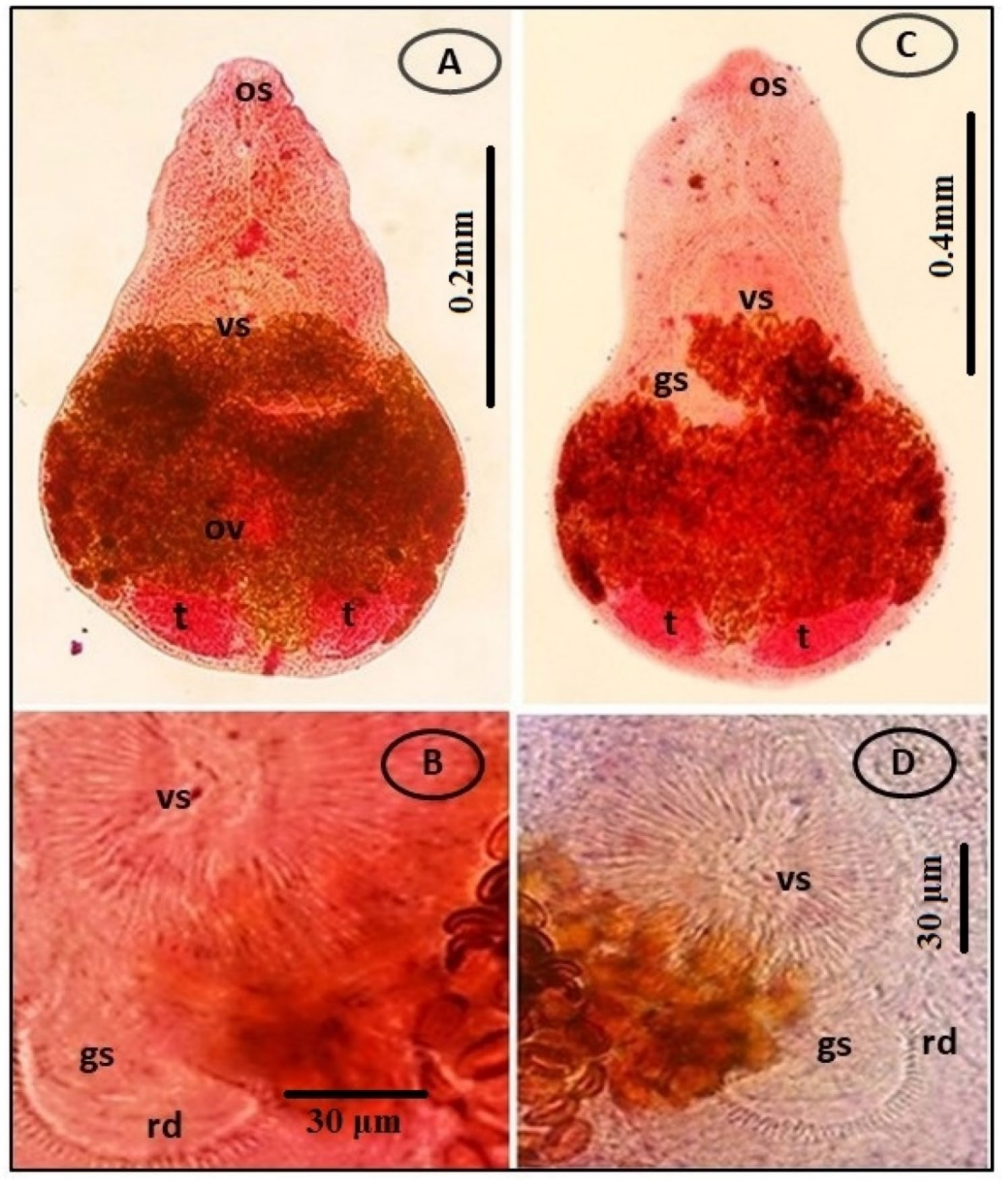
**A**) Photomicrographic of stained specimens of adult flukes of *H. heterophyes***B**) *H. heterophyes* high magnification of armed gonotyl, lying to the dextro-posterior margin of the ventral sucker, armed with more than 70 chitinous spines (rodlets = rd) around the genital sucker (gs) **C**) *H. nocens* shows that oral sucker (os), ventral sucker (vs.) and genital sucker (gs), two horizontal testes (t), ovary (ov). **D**) *H. nocens* high magnification of armed gonotyl, lying to the dextro-posterior margin of the ventral sucker, armed with less than 70 chitinous spines (rodlets = rd) around the genital sucker (gs)

The tissues of *O. niloticus* and *M. cephalus* in Giza and farmed fishes in the Fayoum province and Lake Manzala were simultaneously infected with microscopic live EMC. Based on their morphological features, size, shape, cyst thickness, and presence of pigmentation in the excretory bladder, the tissues were classified into two genera: *Centrocestus* and *Heterophyes*. The experimentally infected pigeons’ small intestines included adult flukes, which were categorized based on their morphological features. The adult flukes were recognized as *C. formosanus*, *H. heterophyes*, and *H. nocens* based on these characteristics (Figs. 3 and 4, and 5).

#### Centrocestus formosanus

##### Encysted metacercaria

It was found that *C. formosanus* EMC was entangled in the gills’ main filaments. With an average size of 152–195 μm (175 ± 15.4) in diameter, the metacercariae have an oval form. The oral sucker is a notable feature in the front part of the metacercarial body and has two rows of spines. The execratory bladder has a distinctive X form. The small esophagus gives way to a broad caecum that extends posteriorly to the excretory vesicle level. At the pharyngeal level, there are two little ocular dark spots (Fig. 3A, B, C, D).

##### Adult flukes

Adult flukes are distinguished by their rounded posterior and tapering anterior bodies, the absence of an oral sucker caudal appendage, and the presence of 30–36 circumoral spines around the sucker arranged in 2 alternative rows. The worms are small truncated measuring 516 × 320 μm in mean. The oral sucker is terminal measuring 57 × 46 μm in mean. The esophagus is short. Ceca was large, bifurcated approximately halfway between ventral and oral suckers, and ended somewhat in front of the ovary. The ventral sucker is smaller than the oral sucker and positioned in the middle of the body. The uterus is short, coiled between the ovary and the seminal vesicle, and contains 5–17 eggs. Vitelline follicles are big and spread laterally from the posterior end to the pharyngeal boundary. Oval eggs with a lattice arrangement on the shell surface, yellowish brown in color, conspicuous operculum (Fig. 3E, F, G).

#### *Heterophyes* species

##### Encysted metacercariae

*Heterophyes* EMC had thick cyst walls, a light greyish color, and a spherical to elliptical shape. The cysts measured 125–153 μm (140 ± 107.8) in diameter. The cyst manifested as a U-shaped formation on its lateral side. The cyst suckers were noticeable, with the ventral sucker being larger than the oral sucker. An oval genital sucker was found behind the ventral sucker on the right lateral side (Fig. 4A, B).

#### Adult flukes

##### Heterophyes heterophyes

The adult flukes are tiny, pyriform, with bodies that range in length from 610 to 890 μm (753 ± 92.7) and width from 426 to 533 μm (484 ± 37). Three different types of sucker were present: a small oral sucker, a large ventral sucker situated median, and a huge sub-median genital sucker equipped with 73–83 chitin rodlets on the gonotyl. Two horizontal testicles positioned side by side and close to the body’s posterior extremities are one of their distinctive morphologies (Fig. 4E A, B).

##### Heterophyes nocens

*H. nocens* adult flukes are elongated, elliptical, and pyriform, 0.82–1.02 mm long (0.93 ± 0.06) and 0.52–0.63 mm wide (0.58 ± 0.04), and it is morphologically close to *H. heterophyes*. The only recognizable difference is the smaller number (52–64) of rodlets on the gonotyl of the genital sucker in comparison to that in *H. heterophyes* (Fig. 5F C, D).

### Molecular identification of the recovered adult flukes

**Fig. 6:**
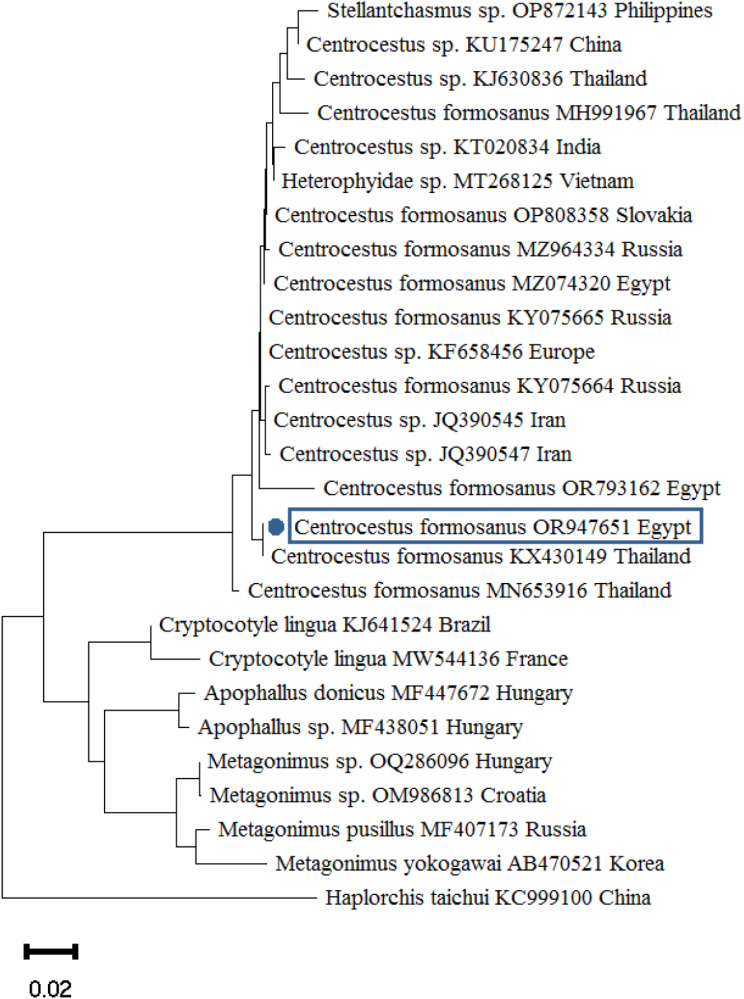
The phylogenetic tree was constructed using the neighbor-joining technique and *C. formosanus* 28 S rRNA region. Blue dots denote the accession numbers for this inquiry. The scale bar at the base of the tree shows how many nucleotide changes have occurred at each location

**Fig. 7:**
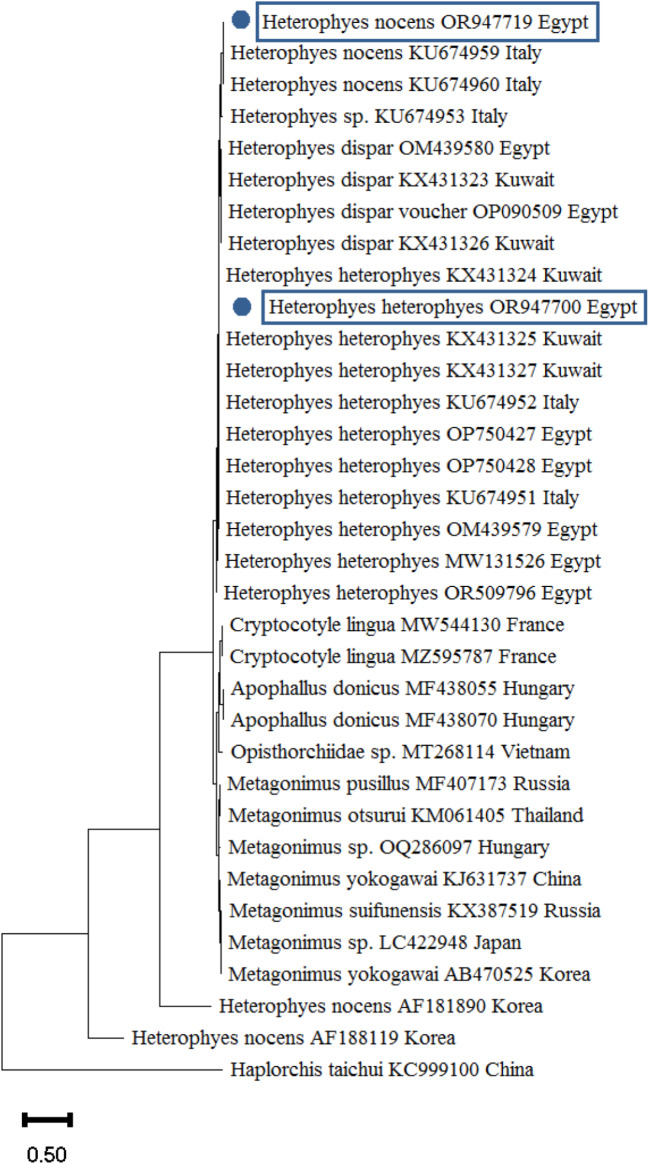
The phylogenetic tree was constructed using the neighbor-joining technique and *H. heterophyes* and *H. nocens* 28 S rRNA region. Blue dots denote the accession numbers for this inquiry. The scale bar at the base of the tree shows how many nucleotide changes have occurred at each location

To reach accurate identification of *Centrocestus* and *Heterophyes* sp. isolated from *O. niloticus* and *M. cephalus*, morphologically identified heterophyid parasites were amplified using PCR where the obtained products were subjected to DNA sequencing. Based on BLAST sequence analysis, the positive PCR results of the 28 S rRNA gene confirmed the heterophyid parasites as *C. formosanus*,* H. heterophyes* and *H. nocens* (Table 3; Figs. 6 and 7). Table (4) listed the accession numbers of the 28 S rRNA gene of *Centrocestus* and *Heterophyes* sp. that were deposited in GenBank.

**Table 4:** The accession numbers of C. Formosanus, H. heterophyes and H. Nocens

Samples	Amplified products (Base pair)	Target gene	Accession number	% of identity
***C. formosanus***	(610)	28 S rRNA	OR947651.1	MZ074320 (100%); KX430149 (99.09%)
***H. heterophyes***	(800)		OR947700.1	KX431325 (100%); MW131526 (99.76%)
***H. nocens***	(1200)		OR947719.1	KU674959 (100%)

### Immunological expression of cytokines

**Fig. 8:**
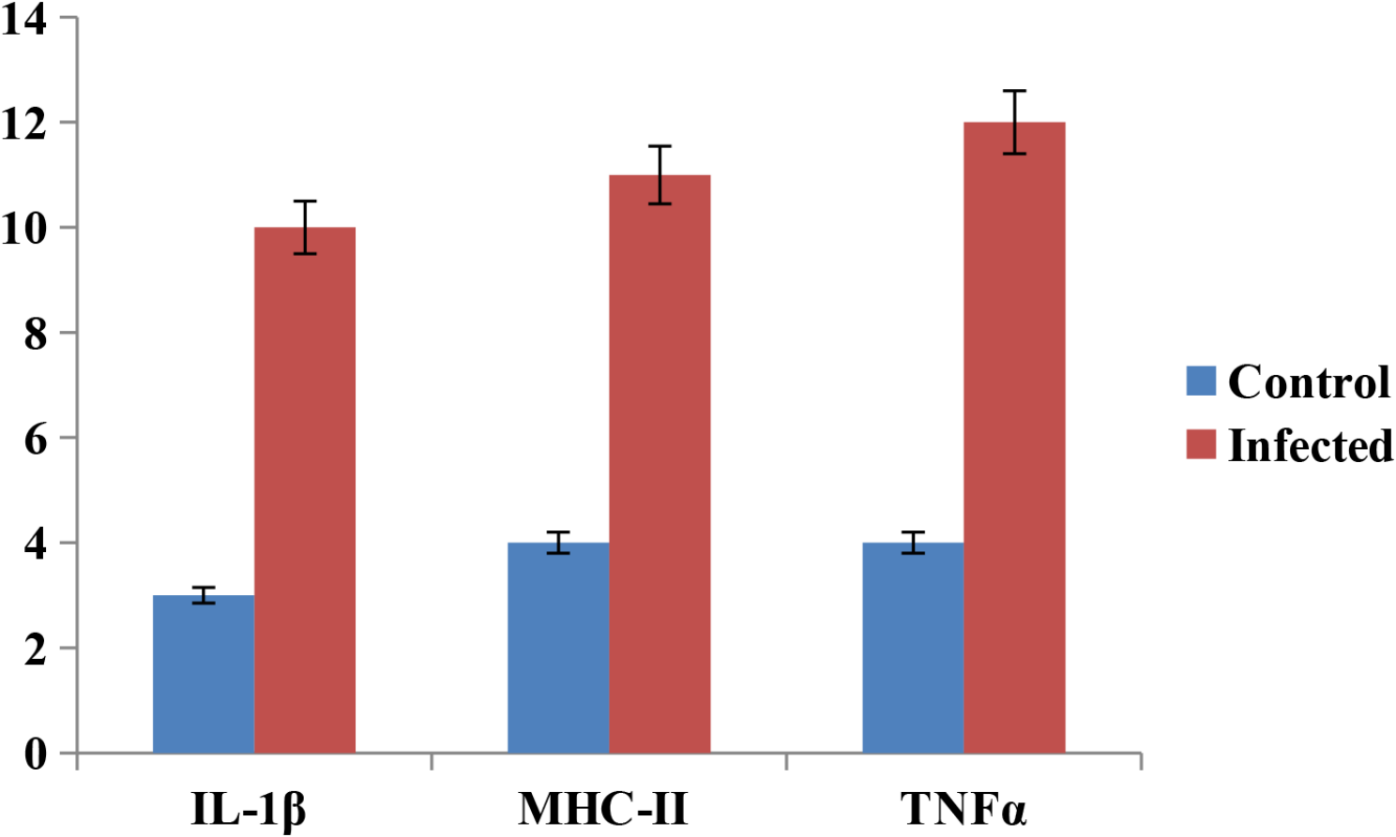
The transcript level of different cytokines in control negative healthy pigeons and *Heterophyes* adult infected pigeons

Samples were categorized as either control or infected intestine; IL-1β was upregulated three times in the control negative samples and ten times in the infected intestine. Moreover. It has been found that MHC-II was upregulated four times in control negative muscles and eleven times in infected samples (Fig. 8). Infected pigeon intestines showed a 12-fold increase in TNF-α transcript levels compared to a 4-fold upregulation in control negative muscles (Fig. 8).

## Discussion

Food-borne zoonotic infections often involve fish-borne zoonotic parasites, which are often endemic in specific regions of the world [35]. Helminths are among the most prevalent infectious agents that have affected and still affect human populations, particularly in developing resource-poor, and marginalized regions of the world. All freshwater fishes typically have them. Fishes serve as an intermediate host for a large number of nematodes, trematodes, and cestodes [11].

In this study, the investigated fishes; *O. niloticus* and *M. cephalus* were found to be concomitantly infected with two types of heterophyid; *Centrocestus* and *H. heterophyus*; *H. nocens* EMC). *Centrocestus* infection was identified in the gills of *O. niloticus* from Giza and Wadi Al-Rayan in the Fayoum province. While heterophyid metacercarial infection *(H. heterophyus* and *H. nocens)* in Lake Manzala’s *M. cephalus.* Furthermore, the overall prevalence of EMCs in the examined fishes was 51.07%. Metacercarial infection in *O. niloticus* from Wadi Al-Rayan and the River Nile was 65% and 51%, respectively. In Lake Manzala, *M. cephalus* metacercarial infection was 33.75%. These results agreed with those found by [2, 3, 36]. The high frequency of these trematodes is usually ascribed to the spread of snails, which act as an intermediate host, and the absence of effective snail control techniques.

Regarding, the prevalence of infection with EMCs differed significantly according to season, summer showed the highest infection rate and winter was the lowest. These findings were the same as those recorded by [1, 19, 37, 38]. The death of cercariae/metacercariae, water quality, and the presence of the first intermediate host could all be factors in the low infection prevalence of EMC during cold seasons [39].

Concerning the distribution of the EMCs in the different regions of the fishes body, the highest number of cysts per gram of tissue was obtained from the tail region of *O. niloticus* and the lowest was in the head region of *M. cephalus.* This result agreed with those of [3, 22, 35, 40]. Additionally, the current research on the spread of the identified EMC species was gathered from the various organs and muscles of fishes that were affected. Each cyst species’ preferred location was identified by this result, which might be attributed to a variety of elements such as the host species, geographic distribution, and genetic diversity of EMC [41]. In the present study, determined EMC belonging to; *Heterophyidae* flukes (*C. formosanus*,* H. heterophyes*, and *H. nocens*). These collected EMC were identified morphologically as described by [6, 14, 42].

The purified amplicons of the 28 S rDNA regions were directly sequenced and submitted to the GenBank under accession numbers: OR947651.1, OR947700.1, and OR947719.1 for *C. formosanus*, *H. heterophyes*, and *H. nocens*, respectively. BLAST analysis of *C. formosanus* revealed strong similarity with [43–45] in Thailand, Egypt, and Vietnam when comparing the retrieved sequences with other *Heterophyidae*, respectively. However, *H. heterophyes* BLAST analysis revealed nucleotide similarities with that of [46, 47] from Kuwait and Italy, respectively. Regarding *H. nocens*, it demonstrated a significant degree of nucleotide similarity with both [46, 48] in Korea and Italy, respectively.

Furthermore, the present investigation is concerned with the demonstration of the immunological bioassay of pigeons rather than rats as its value for food consumers as a well-appreciated source of protein and has an important role in the biological life cycle of different EMC as second intermediate host as reported by [15, 49]. Other studies have shown that the development of the host’s defense mechanisms against parasites is aided by the stimulation of humoral host immune responses. The current investigation’s findings indicate that the various cytokines rapidly rose in the infected pigeon’s intestines. Additionally, the infection expanded due to the parasite’s ejection from the host and the host’s clinical affliction, both of which induced humoral immune responses. The results were consistent with up-regulation in other helminthic infections, as recorded by [50–53].

## Conclusion

Heterophyiasis is a highly endemic disease in the Nile Delta, Egypt where the habit of consuming raw or inadequately cooked *O. niloticus* and *Mugil cephalus*. Pigeons and rats can play an important part in fish-borne zoonotic trematode transmission since they serve as a natural and experimental host. This study aimed to update the epidemiological information and morphological description of two distinct heterophyid metacercariae in Egypt: Giza, Wadi Al-Rayan, and Lake Manzala those areas are linked with different types of heterophyid infection. The first genetic descriptions of three heterophyid adult fluke species in Egypt were validated by GenBank: *C. formosanus*,* H. heterophyes*, and *H. nocens*, with accession numbers OR947651.1, OR947700.1, and OR947719.1, respectively. Additionally, studies were conducted using the initial examination of cytokines and host immune responses following heterophyid species infection in Egyptian pigeons. According to this study, more investigation is necessary to find other trematode species, especially those that are important to zoonoses. We suggest controlling rats, snails, and migrating birds in addition to thoroughly boiling and inspecting fishes to destroy metacercariae.

## Data Availability

All the authors declare that all the data supporting the results reported in our article were included in this article. The datasets generated or analyzed during the current study are available in the GENEBANK repository, Centrocestus formosanus, Heterophyes heterophyes, and Heterophyes nocens, with accession numbers OR947651.1, OR947700.1, and OR947719.1, respectively.
